# Facilitators and barriers to implementing antimicrobial stewardship programs in public South African hospitals

**DOI:** 10.1017/ash.2022.355

**Published:** 2023-02-23

**Authors:** Lodewyk Nicolaas Scheepers, Christina Maria Niesing, Petra Bester

**Affiliations:** The Africa Unit for Transdisciplinary Health Research (AUTHeR), Faculty of Health Sciences, North-West University, Potchefstroom, North West Province, South Africa

## Abstract

**Objective:**

The South African National Department of Health released guidelines and recommendations for antimicrobial stewardship (AMS) programs to be established in public healthcare facilities. Their implementation remains challenged, especially in North West Province, where the public health system functions under severe strain. This research explored and interpreted the facilitators that strengthen and barriers that hinder the implementation of the national AMS program in public hospitals in North West Province.

**Design:**

A qualitative design and interpretive descriptive approach enabled insight into the realities of AMS program implementation.

**Setting:**

Public hospitals in North West Province, sampled through criterion sampling (n = 5).

**Participants:**

Purposive criterion sampling of healthcare practitioners (n = 30) actively participating in AMS programs in the 5 sampled public hospitals.

**Method:**

Qualitative, interpretive description with semi-structured individual interviews that were digitally recorded and transcribed. The ATLAS.ti version 8 software facilitated content analysis, followed by second-level analysis.

**Results:**

In total, 4 themes, 13 categories, and 25 subcategories emerged. We detected dissonance between government AMS ideals and the realities of AMS program implementation in public hospitals. A multilevel AMS leadership and governance vacuum exists in a dysfunctional health ecosystem in which AMS must operate. Healthcare practitioners agreed on the importance of AMS despite different understandings of AMS and ineffective multidisciplinary teams. Discipline-specific education and training are essential for all AMS participants.

**Conclusions:**

AMS is essential yet complex, and its contextualization and implementation are underestimated in public hospitals. Recommendations are focused on a supportive organizational culture, contextualized AMS program implementation plans, and changes in management.

The inappropriate use and overuse of antimicrobials may lead to antibiotic resistance, adversely affecting morbidity, mortality, and healthcare costs.^1^ Low- and middle-income countries such as South Africa are not exempt from this problem,^2^ and surveillance data confirm increased resistance of pathogens that cause severe infections with limited treatment options, particularly gram-negative organisms.^3,4^ The South African National Department of Health responded to this threat by proposing its Antimicrobial Resistance National Strategic Framework (ARNSF) for 2014–2024, comprising 5 interconnected objectives to minimize antimicrobial resistance. Emphases are placed on antimicrobial stewardship (AMS) and developing and implementing a national AMS program.^5^

An effective AMS program is recognized globally as an essential strategy to counter antimicrobial resistance and refers to a synchronized set of interventions created to improve the responsible use of antimicrobials. AMS requires a multidisciplinary approach that improves patient outcomes by promoting the selection of the optimal antimicrobial regimen, dose, duration of therapy, and route of administration.^6^ Successful AMS programs ensure that antimicrobials are used most effectively to limit the emergence of resistant pathogens while ensuring patient safety.^7^ However challenging the implementation of AMS in high-income countries is, it is even more so in low- and middle-income countries.^8^

Although interventions used by AMS programs are well described in international guidelines,^9,10^ data concerning the practical implementation of these interventions in Africa are limited.^8^ An earlier international study by Howard et al^11^ identified a lack of AMS programs on the African continent. It highlighted the differences in barriers to implementing AMS in Africa compared with North America and Europe. These challenges and the lack of data on the successful implementation of AMS programs in public hospitals confirm that AMS models from high-income countries are not translatable to South Africa.^2^ Also, no study has been conducted on the comprehensive barriers and challenges to implementing AMS in North West Province’s dichotomous health system,^11,12^ nor the complexities of the context. A qualitative, interpretive design enabled an in-depth understanding^13^ of the facilitators and barriers to AMS program implementation in public hospitals in North West Province. An interpretive description^13^ was ideal for synthesizing healthcare professionals’ theoretical and practical knowledge within public hospitals about the implementation of AMS and articulating the results to inform the “actual doing” in practice.

## Methods

The North West Department of Health delivers public healthcare to nearly 7% of the national population residing across a rural geographical spread. It operates with facilities that make up 8.7% of the total number of facilities in the country.^12^ Most of these facilities are old and inequitably distributed and face the significant challenges of large patient numbers, a shortage of personnel, and the quadruple burden of HIV/AIDS, tuberculosis (TB), noncommunicable diseases, and maternal-, newborn-, and child-related diseases.^4,12,14^ Ethics approval (certificate no. NWU-00115-18-S1) and hospital permissions were obtained for the study. South Africa’s hospitals are divided into district, regional, tertiary-care, central, and specialized hospitals. Criterion sampling was used to select 5 public hospitals in North West Province^15^ that could implement AMS. The selected hospitals included tertiary-care and regional public hospitals.

Purposive, quota sampling of healthcare practitioners actively involved with AMS in these facilities was conducted, fulfilling other inclusion and exclusion criteria.^15,16^ The gatekeepers (hospital chief executive officers and district pharmacists) facilitated the researcher’s access to the hospitals, and mediators facilitated participant recruitment.^17^ Participants gave written informed consent. The researcher, a pharmacist, conducted semistructured individual interviews at each hospital with registered nurse practitioners (n = 9), pharmacists (n = 12), and medical practitioners (n = 9) on facilitators and barriers to AMS program implementation in their hospitals. Regarding sex, 53% of the sample were women, and 47% were men. Most participants (77%) were aged 25–45 years (Table 1). Participants were professionally registered and had bachelor’s degrees as a minimum qualification. Digitally voice-recorded interviews were transcribed verbatim. Transcripts were organized and sorted, and the data were analyzed following the thematic analysis steps of Creswell et al,^18^ supported by ATLAS.ti version 8 software (ATLAS.ti, Berlin, Germany). Secondary data analysis followed.

**Table 1.** Demographic Characteristics of the Participants

Profession	Level of Seniority/Education (n = 30)	Sex		Ethnicity			Age, Years			
		Male	Female	African	White	Indian	25–35	36–45	46–55	56–65
Doctors	Physician	2		1		1				2
	Specialist (pediatrician)	1		1					1	
	Clinical manager	2	1	3				3		
	Head of department	2		2				1	1	
	Medical officer	1		1				1		
Pharmacists	Pharmacy manager	1	2	1	2			2	1	
	Ward pharmacist	2	4	1	4	1	5	1		
	Pharmacist	1	2		2	1	2	1		
Nurse practitioners	Nurse manager		1	1				1		
	Ward nurse	2	2	4			1	2	1	
	Infection control nurse		4	2			1	2	1	
Total		14	16	17	10	3	9	14	5	2

## Results

**Fig. 1.**
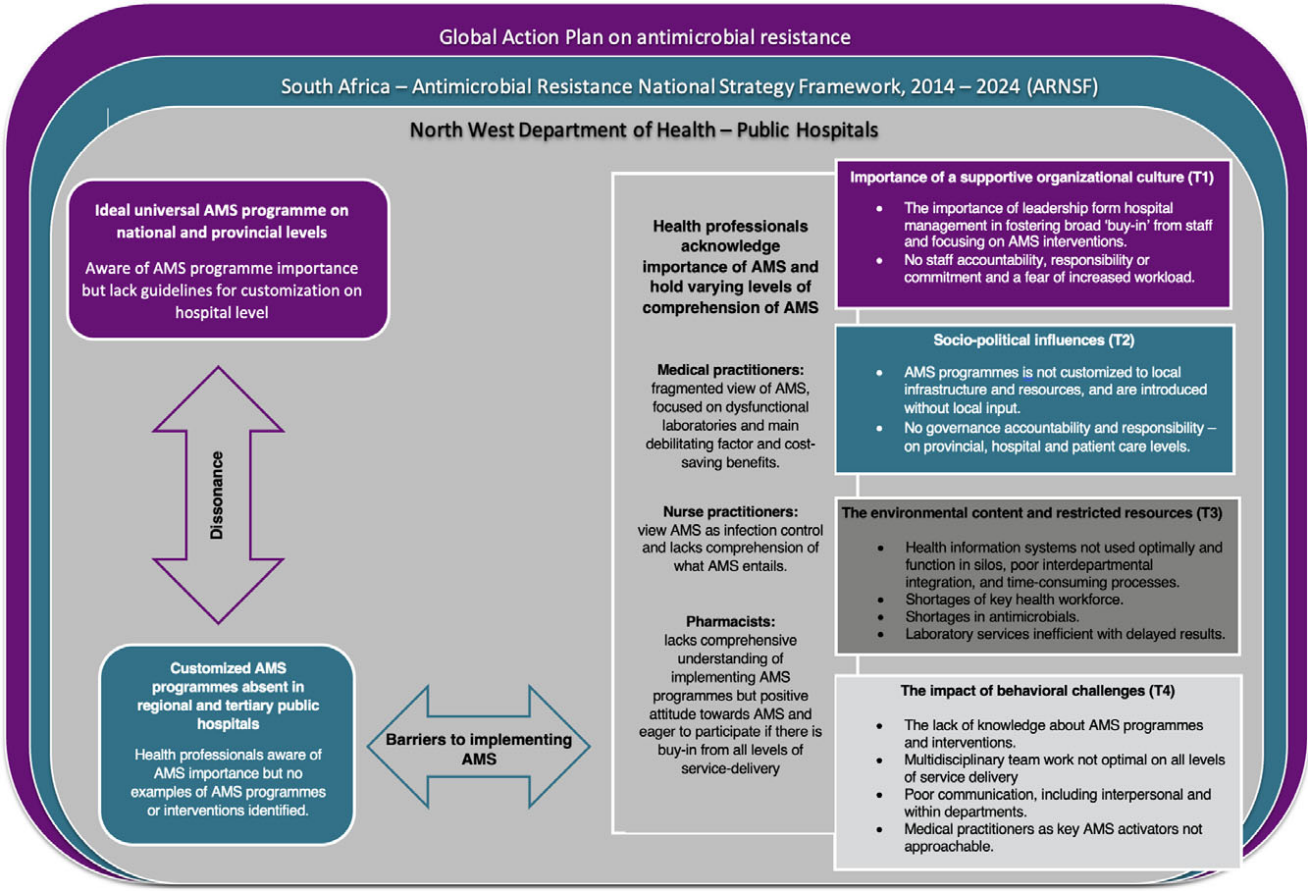
Conceptual framework of the realization of AMS programs within public hospitals in North West Province. Note. AMS, antimicrobial stewardship; WHO, World Health Organization.

True to the interpretive descriptive design, the results (4 themes, 13 categories, and 25 subcategories) were organized into a conceptual framework (Fig. 1 and Table 2). This framework depicts the relationships between the 4 themes and presents an interpreted understanding of the realities of AMS program implementation in public hospitals in North West Province. The conceptual framework is not a model and cannot be implemented or tested.

**Table 2.** Summary of Data Obtained From the Interviews

Theme	Categories	% of Respondents Who Agree/Strongly Agree With Each Statement		
		Doctors (N = 9)	Pharmacists (N = 12)	Nurses (N = 9)
Importance of a supportive organizational culture	Buy-in from staff	55.6	50.0	11.1
	Leadership engagement	33.3	58.3	55.6
	Accountability, responsibility, and commitment	55.6	33.3	11.1
Social-political influences	Universal structure	44.4	58.3	22.2
	Structure from the top down	66.7	33.3	33.3
	Lack of leadership from the Department of Health	66.7	58.3	11.1
The environmental context and restricted resources	Human resources	44.4	25.0	77.8
	Health information technology and data analysis resources	77.8	58.3	44.4
	Lack of laboratory services	88.9	75.0	33.3
	Limited availability of antimicrobials	33.3	41.7	33.3
The impact of behavioral challenges	Educations and training	22.2	41.7	22.2
	Cooperation and leadership	44.4	50.0	11.1
	Communication	66.7	33.3	33.3
	Behavioural change	33.3	83.3	55.6

We detected dissonance between the universal AMS program ideal presented by the National Department of Health’s ARNSF for adoption in each province and the realities of AMS in the participating hospitals. Despite a national requirement for AMS programs in public hospitals described in the ARNSF, actual AMS programs were absent in the selected public hospitals. Participants could not provide examples of AMS programs, projects, interventions, or initiatives. The ideal AMS program proposed by the National Department of Health and based on the Infectious Diseases Society of America (IDSA) guidelines recommends establishing an AMS team comprising various experts, including infectious disease specialists, clinical microbiologists, and pharmacists trained in AMS. However, North West Province public hospitals function without these specialized health professionals.

The different types of health professionals acknowledged the importance of AMS but held different understandings of it. The medical practitioners had a fragmented perspective of AMS and focused on patient outcomes, cost-saving benefits, and the impact of dysfunctional laboratory services. The nurse practitioners perceived AMS as similar to infection control without understanding its critical role in an AMS program. Although the pharmacists lacked a comprehensive understanding of AMS program implementation strategies, they seemed to possess a natural inclination to implement AMS interventions and acknowledged the importance and absence of multidisciplinary support and managerial buy-in.

AMS program implementation is absent in these public hospitals owing to a multilevel AMS leadership and governance vacuum. Despite a national AMS program agenda and practical guide to AMS intended to serve as a blueprint, leadership for such programs was absent at provincial, hospital, and patient-care levels. In the absence of AMS leadership and governance, there was a generalized lack of responsibility, accountability, and commitment to AMS. Health professionals perceived AMS as a top-down and invasive initiative; they generally preferred to remain AMS followers and not take the lead. Without infectious disease specialists and clinical microbiologists, the interviewed health professionals proposed that hospital clinical managers take the lead.

Health professionals acknowledged that implementing an AMS program was essential, yet the medical and nursing practitioners feared additional workload without sufficient support in the absence of an AMS governance structure. The health professionals agreed that education and training could strengthen AMS program implementation. Medical and nursing practitioners voiced the need for training on the clinical management of AMS interventions. Pharmacists, however, proposed training on practical AMS program implementation.

Dysfunctional health ecosystems hinder the implementation of a universal AMS program in these public hospitals. Health professionals explained that AMS programs could not be implemented in an overburdened health system with shortages in the health workforce. Staff shortages were identified as relating to general medical practitioners, nursing staff, and AMS key role players such as clinical microbiologists.

Although digital health systems exist in units, departments, and laboratories, these systems function in silos, causing health professionals to perform time-consuming and repetitive paperwork. The 5 public hospitals have not adopted existing digital health solutions to an equal extent. Furthermore, health professionals reported resource constraints and stockouts of specific AMS-required antimicrobials. In resource-restricted settings, health professionals are unable to prescribe appropriate treatment. Also, laboratories cannot respond promptly to requests and local infection patterns. There are shortages in laboratory materials, and culture and susceptibility results are not received in time.

Finally, ineffective multidisciplinary teamwork and communication obstruct the implementation of AMS programs. Ineffective multidisciplinary collaboration is present at hospital and patient-care levels. Medical practitioners are perceived as the activators of treatment regimens. Still, they are considered less approachable by pharmacists and nurse practitioners because of ineffective interpersonal communication within and between different units and departments.

## Discussion

In this research, we investigated barriers to and facilitators of implementing a national AMS program in North West Province’s public health sector, specifically public hospitals. However, our findings highlight the discord between the AMS programs, according to the National Department of Health’s ARNSF and actual AMS interventions in the hospitals.^19–21^

The major themes discerned from this qualitative study were sociopolitical influences, limited resources, the importance of a supportive organizational culture, and the impact of behavioral challenges. More specifically, subthemes that emerged were lack of leadership, accountability, communication, and education.

*Theme 1: Importance of a supportive organizational culture*

The 3 groups of respondents who agreed with the statements varied significantly (Table 3). This variation indicated that the 3 groups of respondents had various viewpoints on the statements. Previous findings from academic hospitals in the United States demonstrated that a supportive organizational culture and leadership engagement is essential for a successful antibiotic stewardship program.^22^ Furthermore, the suggested models should accommodate the local healthcare infrastructure, organizational culture, and available resources, and, most importantly, they must have buy-in from all healthcare practitioners tasked with AMS.^11^ Although they are naïve about AMS program implementation, pharmacists are generally the healthcare practitioners most eager to implement these programs. They understand that buy-in from all staff at all levels of the hospital structure is essential.^23^ Much has been reported about the clinical role of pharmacists and their key responsibilities in monitoring, implementing, and providing feedback for AMS activities.^20,24–26^ This role is even more relevant in an alternative model in which healthcare practitioners are not specially trained in infectious diseases, as is the case in North West Province.^23^ Buy-in from prescribers and nursing practitioners is gained through effective communication and requires changes in individual behavior and organizational processes.^25^

**Table 3.** Theme 1: Importance of a Supportive Organizational Culture

Importance of a Supportive Organisational Culture			
Categories and Subcategories	Proportion (%) of Respondents Who Agree/Strongly Agree With Each Statement		
	Doctors (N = 9)	Pharmacists (N = 12)	Nurses (N = 9)
**Buy-in from staff**	55.6	50.5	11.1
The importance of leadership in fostering broad ‘buy-in’ and commitment			
**Leadership engagement**	33.3	58.3	55.6
The lack of leadership from hospital management			
The importance of leadership in engaging staff and focusing on AMS interventions			
**Accountability, responsibility, and commitment**	55.6	33.3	11.1
Who takes accountability and responsibility to implement AMS programs			

With more significant input from staff and a desire to see their facility succeed, a flatter governance structure is an essential enabler of AMS.^24^ Broad acceptance of AMS throughout institutions starts with a clear vision, commitment, and support from senior healthcare leaders and the institutional administration.^22–24^ The Centers for Disease Control (CDC) observes that support from institutional leadership is critical to the success of any AMS program. As such, it recommends hospital leadership commitment as the first core element of AMS.^9^ However, our findings suggest a general gap in the leadership and governance of AMS in public hospitals.^22^ This poor leadership exists on all healthcare levels: provincial, hospital, and patient care.^19^ Although the South African ARNSF describes a comprehensive approach to tackling antimicrobial resistance, with clear leadership roles and responsibilities,^20^ our research revealed deficits in responsibility, commitment, and, especially, accountability toward AMS.

*Theme 2: Sociopolitical influences*

Doctors and pharmacists agreed more with the statements than the nurses (Table 4). In countries such as India and Burkina Faso, the national AMS guidelines were not fit to the local context and were thus implemented unsuccessfully.^27^ Implementing AMS activities and approaches is more likely to succeed if local data on susceptibility and resistance patterns influence prescribing behavior.^28^ In North West provincial hospitals, a lack of engagement and consultation led to healthcare practitioners perceiving AMS as a top-down event and being cautious of increasing their workload without organizational support. Clinicians perceive the national AMS program as a universal structure that cannot account for the local healthcare structure, geography, culture, and behavioral determinants of resources.^11^

**Table 4.** Theme 2: Sociopolitical Influences

Sociopolitical Influences			
Categories and Subcategories	% of Respondents Who Agree/Strongly Agree With Each Statement		
	Doctors (N = 9)	Pharmacists (N = 12)	Nurses (N = 9)
**Universal structure**	44.4	58.3	22.2
AMS program that is not customised to local infrastructure and resources			
**Structure from the top down**	66.7	33.3	33.3
The AMS program was introduced without local input.			
**Lack of leadership from the Department of Health**	66.7	58.3	11.1
There is no responsibility or accountability for implementing the AMS program from the provincial Department of Health.			

AMS also requires commitment and leadership at the government level and willingness to deliver set goals at the point of health care.^28^ Government health departments are tasked with implementing AMS and need to plan program implementation carefully. At the same time, they must sell the program to its grassroots stakeholders (eg, clinicians, nurses, pharmacists, and infection control managers) driving the operational component at all levels.^28,29^ As indicated from our research, implementing an AMS program is impossible without a clear commitment from leaders and policy makers as well as buy-in from all healthcare providers.^30^

*Theme 3: The environmental context and restricted resources*

Regarding the environmental context and restricted resources, the nurses strongly agreed with the statements on human resources as opposed to the doctors and pharmacists, that agreed more with the statements on health information technology and data analysis resources and the lack of laboratory services (Table 5). Implementing AMS programs is even more challenging when resources, such as personnel, laboratory services, antibiotics, and funding, are limited.^8,20,31,32^ Participants in our research reported similar challenges and considered the lack of resources a major limitation to implementing AMS programs.^27^

**Table 5.** Theme 3: The Environmental Context and Restricted Resources

The Environmental Context and Restricted Resources			
Categories and Subcategories	% of Respondents Who Agree/Strongly Agree with Each Statement		
	Doctors (N=9)	Pharmacists (N=12)	Nurses (N=9)
**Human resources**	44.4	25.0	77.8
The availability of key personnel (eg, clinical microbiologists)			
Shortage of staff, especially nursing practitioners and clinicians.			
**Health information technology and data analysis resources**	77.8	58.3	44.4
The AMS interventions are time-consuming.			
Problems with data and information systems and resources to utilize it			
Clinical data on the current use of antimicrobials at each institution are not available.			
**Lack of laboratory services**	88.9	75.0	33.3
Communication from the laboratory about requests and local infection patterns			
The shortage of laboratory services and materials			
Receiving culture and susceptibility results in time			
**Limited availability of antimicrobials**	33.3	41.7	33.3
Shortages in the supply of antibiotics			

A lack of information technology has been ranked as the primary barrier to implementing successful programs on the African continent.^11^ The time-consuming completion of written requests in the absence of an integrated digital health system and a lack of information technology integration between pharmacy, laboratory, and hospital wards have resulted in poor access to patient information.^31^

Not only was AMS seen as increasing the workload of clinical staff, but no reports were generated to track interventions and outcomes; hence, local data are not reported to administrators and the provincial health department. As mentioned in the ARNSF, these reports are crucial for the National Department of Health to plan and introduce strategies^5^ to curb environmental context-specific barriers, such as the limited availability of antimicrobial options in hospitals where multidrug-resistant bacteria are prevalent.^31^

Raw material and medicine shortages are an increasing challenge worldwide and also apply to North West Province, where lack of access to essential antimicrobials is more problematic than their excessive and inappropriate use.^6^

The literature confirms that an effective AMS program depends on a multidisciplinary team approach in which essential collaboration between units and departments exists.^10,22^ In well-resourced settings such as the United States and Europe, multidisciplinary teams often include trained AMS and infection specialists such as infectious disease physicians, clinical pharmacists, and microbiologists.^20^ However, in North West Province, hospitals often have to function without these specialities.^1,2^ Health workforce shortages hinder the implementation of AMS programs,^31^, especially in the South African public healthcare sector; therefore, the use of existing human resources, including nonspecialized pharmacists, registered nurses, and other healthcare members, is essential.^6^

The microbiology laboratory’s role goes well beyond the reporting of culture and susceptibility of individual patients and is fundamental in guiding prescriber behavioral change.^11,29^ Every clinician in our research touted weak laboratory infrastructure and delayed result reporting as significant stumbling blocks to implementing AMS. As such, participants acknowledged that they did not send specimens or did not follow-up on the results. Literature reports and participants reiterated ineffective communication from laboratories and lack of laboratory resources as significant barriers to implementing AMS.^30,31^

*Theme 4: The impact of behavioral challenges*

Doctors mostly agreed with the statements made on communication in the impact of behavioral challenges (Table 6). Nurses mostly agreed with the statements made on behavioral change in contrast to pharmacists, who mostly agreed with the statements on cooperation and leadership and behavioral change. Brink et al^2^ studied the impact of AMS interventions in South Africa and noted that the significant challenge in changing organizational culture and prescribing practices lies in the public healthcare sector.^21^ Notable barriers to implementing the national AMS program in this sector include a shortage of healthcare professionals with the expertise to lead and coordinate AMS programs, poor communication, and inadequate education and training, compounded by geographical disparity.^2,22,31^ Based on a careful assessment of these challenges, the opportunity to enhance the effects of AMS programs through behavioral approaches is immense.^31^ AMS activities require changes in individual behavior (eg, cooperation and leadership) and organizational processes (eg, communication).^25^ The crucial roles of the science and skills of behavior improvement beyond infectious diseases and microbiology cannot be underestimated if the effective and sustainable implementation of AMS programs is to be achieved.^23^ Not only do interprofessional processes, such as communication, cooperation, and leadership among all members of the AMS team, influence the implementation of AMS programs, a lack of willingness to change behavior hinders implementation efforts.^31^

**Table 6.** Theme 4: The impact of behavioral challenges

The Impact of Behavioral Challenges			
Categories and Subcategories	Proportion (%) of Respondents Who Agree/Strongly Agree With Each Statement		
	Doctors (N = 9)	Pharmacists (N = 12)	Nurses (N = 9)
**Education and training**	22.2	41.7	22.2
The lack of knowledge about AMS programs and interventions			
The lack of knowledge of current use of antibiotics			
Medical professionals lacking relevant skills for specific AMS interventions			
**Cooperation and leadership**	44.4	50.0	11.1
Establishing a multidisciplinary team with representatives from each department			
Currently, professions work in silos and fragment interprofessional collaboration			
Leading the AMS team and coordinating AMS interventions			
**Communication**	66.7	33.3	33.3
Poor communication (including interpersonal) within departments			
**Behavioral change**	33.3	83.3	55.6
Clinicians’ resistance and lack of willingness to change			
Perceived unhelpful attitudes of clinicians			

Note. AMS, antimicrobial stewardship.

One of the most basic and valuable tools is the provision of education opportunities that introduce AMS interventions to clinicians and other healthcare professionals and inform them about their implementation.^10,32^ Literature confirms that healthcare practitioners agree on the importance of AMS but have a fragmented understanding of what an AMS program truly entails.^20,28^ This is especially true in the South African public healthcare environment, where a lack of coordinated standardized training programs for AMS has been identified as a major barrier.^20^ However, neither the literature nor our research findings could confirm the different needs of pharmacists, clinicians, and nursing practitioners regarding AMS education and training.

In conclusion, implementing AMS programs in public hospitals in North West Province is complex. AMS programs are essential, and their operationalization is underestimated. Despite national buy-in for AMS program implementation and sporadic training initiatives, these public hospitals display a dire paucity of AMS programs. The lack of accountability, responsibility, and commitment from the hospital and provincial health department leadership and sociopolitical influences hamper the implementation of AMS and healthcare service delivery. The key learning from the findings concerns the environmental context and relates to the lack of resources.

Public hospitals lack the clinical AMS specialists indicated in the national AMS program, and available healthcare practitioners are expected to implement AMS despite a fundamental understanding of what an AMS program entails (Table 7). The limited availability of antimicrobials aggravates the challenges associated with lacking human resources, information technology, and laboratory services. However, this research underlines the value of behavioral change. AMS program implementation requires training and education adapted to different types of healthcare practitioners and focused on behavioral change. In-service awareness and skills training must facilitate multidisciplinary team dynamics and communication.

**Table 7.** Summary of Major Barriers and Enablers for Implementing AMS Program in Regional and Rural Hospitals

Major Barriers	Major Enablers
Lack of resources	Although lacking, education and training on AMS are acknowledged
Lack of accountability and responsibility at provincial and institutional level	Positive communication within healthcare facilities
Inadequate laboratory services or clinical microbiology support	Positive attitudes, commitment, and pride in the local facilities
Lack of internal expertise within healthcare facilities and leadership at provincial level	
Preserving the national AMS as a universal top-down structure	
Ineffective communication from provincial and national level	

A strategy focused on building a supportive and cohesive organizational culture may strengthen AMS programs contextualized to improve healthcare outcomes. A multilevel implementation plan that acknowledges the available healthcare practitioners, with additional training per hospital, is proposed. This plan will address leadership and governance gaps and facilitate buy-in based on change management.

The multilevel implementation plan should also include (1) strengthening the health ecosystem by addressing the shortages of key health staff that can contribute to AMS; (2) integrating digital health information systems to optimize requests for and reporting in AMS within a reasonable time and minimize additional paperwork; (3) targeting inventory management to enhance the availability of specific antimicrobials; and (4) optimizing laboratory services through the availability of resources specific to AMS. However, the first step critical to implementing AMS in North West Province is engaging with government, policy makers, and healthcare leaders to obtain adequate support.
